# Alcohol consumption and risk of dementia up to 27 years later in a large, population-based sample: the HUNT study, Norway

**DOI:** 10.1007/s10654-015-0029-2

**Published:** 2015-05-13

**Authors:** Ellen Melbye Langballe, Helga Ask, Jostein Holmen, Eystein Stordal, Ingvild Saltvedt, Geir Selbæk, Arvid Fikseaunet, Sverre Bergh, Per Nafstad, Kristian Tambs

**Affiliations:** Division of Mental Health, Norwegian Institute of Public Health, P.O. Box 4404, 0403 Nydalen, Oslo, Norway; Norwegian National Advisory Unit on Ageing and Health, Vestfold Hospital Trust, Tønsberg, Norway; HUNT Research Centre, Department of Public Health and General Practice, Norwegian University of Science and Technology (NTNU), Levanger, Norway; Namsos Hospital, Nord-Trøndelag Health Trust, Namsos, Norway; Department of Neuroscience, Norwegian University of Science and Technology (NTNU), Trondheim, Norway; Department of Geriatrics, St Olav Hospital, University Hospital of Trondheim, Trondheim, Norway; Centre for Old Age Psychiatric Research, Innlandet Hospital Trust, P.O. Box 68, 2312 Ottestad, Norway; Levanger Hospital, Nord-Trøndelag Health Trust, Levanger, Norway; Institute of Health and Society, University of Oslo, Pb 1130 Blindern, 0318 Oslo, Norway; Akershus University Hospital, Lørenskog, Norway; Division of Epidemiology, Norwegian Institute of Public Health, P.O. Box 4404, 0403 Nydalen, Oslo, Norway

**Keywords:** Dementia, Risk factor, Alcohol consumption, Epidemiology, Longitudinal study

## Abstract

The relationship between alcohol consumption and dementia risk is unclear. This investigation estimates the association between alcohol consumption reported in a population-based study in the mid-1980s and the risk for dementia up to 27 years later. The entire adult population in one Norwegian county was invited to the Nord-Trøndelag Health Study during 1984–1986 (HUNT1): 88 % participated. The sample used in this study includes HUNT1 participants born between 1905 and 1946 who completed the questionnaire assessing alcohol consumption. A total of 40,435 individuals, of whom 1084 have developed dementia, are included in the analysis adjusted for age, sex, years of education, hypertension, obesity, smoking, and symptoms of depression. When adjusting for age and sex, and compared to reporting consumption of alcohol 1–4 times during the last 14 days (drinking infrequently), both abstaining from alcohol and reporting consumption of alcohol five or more times (drinking frequently) were statistically significantly associated with increased dementia risk with hazard ratios of 1.30 (95 % CI 1.05–1.61) and 1.45 (1.11–1.90), respectively. In the fully adjusted analysis, drinking alcohol frequently was still significantly associated with increased dementia risk with a hazard ratio of 1.40 (1.07–1.84). However, the association between dementia and abstaining from alcohol was no longer significant (1.15, 0.92–1.43). Equivalent results for Alzheimer’s disease and vascular dementia indicated the same patterns of associations. When adjusting for other factors associated with dementia, frequent alcohol drinking, but not abstaining from alcohol, is associated with increased dementia risk compared to drinking alcohol infrequently.

## Introduction

Excessive alcohol consumption may directly or indirectly increase the risk for dementia [1–4]. Conversely, it is assumed that light to moderate alcohol consumption may have favourable cardiovascular effects, which in turn are associated with reduced dementia risk. Several studies have found that light to moderate alcohol consumption may be associated with decreased dementia risk compared to abstaining from alcohol [5–7].

With one exception [8], the approximately 25 studies that so far have specifically investigated the association between alcohol consumption and dementia risk [9] include only individuals aged 65 years or older at the time of the alcohol consumption assessment [3, 10–18]. Hence, the findings are only generalisable to alcohol consumption close in time to dementia onset. Moreover, most studies investigating the association between alcohol consumption and dementia risk involve study periods of less than 10 years [7]. Dementia may gradually develop over several years. The shorter the time span between the exposure assessment (i.e., alcohol consumption) and outcome assessments (i.e., dementia), the more difficult it is to interpret whether estimated statistical associations are causal or reflect reverse causation.

The aim of this study was to investigate the dementia risk associated with self-reported alcohol consumption using a large population-based sample while adjusting for age, sex, education, hypertension, BMI, smoking, and symptoms of depression. Alcohol consumption was assessed at baseline in 1984–1986, up to 27 years prior to the identification of dementia diagnosis.

## Methods

### Study design and participants

Information about self-reported alcohol consumption and other health and lifestyle variables was obtained from the Nord-Trøndelag Health study (HUNT). Data on dementia diagnoses were obtained from The Health and Memory Study (HMS). Registry data on education and date of death were obtained from Statistics Norway (SN). The three data sets were linked using the personal identification number assigned to all Norwegian citizens as the matching key. This number was removed for privacy reasons before the linked dataset was made available to the researchers.

Both the HUNT and HMS were conducted in the county of Nord-Trøndelag (NT) in Norway. In 2003 the adult NT population consisted of around 94,000 adults considered fairly representative of the general Norwegian population in terms of geography, economy, industry, and age distribution [19]. The HUNT study, composed of three extensive health examinations, is described in detail elsewhere [19, 20]. In 1984–1986, the entire NT population aged 20 years or older, a total of 85,100 persons, were invited to participate in HUNT1. The participants underwent medical examinations (measurement of blood pressure, height, and weight), and they completed self-administered questionnaires, including questions about medical history and lifestyle. The participation rate in HUNT1, including both the medical examination and a questionnaire (Q1) was 88 % (N = 74,997). Of the invited subjects 76 % (N = 64,543) also completed and returned a second questionnaire (Q2), which was taken home after the medical examination and returned by mail. Of the individuals invited to HUNT1, 72 % (N = 61,553) provided a valid response to the alcohol frequency consumption item in Q2 [20].

The original data file comprised a total of 106,485 people participating in one or more of the three waves of HUNT data collections. Individuals under 65 years of age are rarely diagnosed with dementia. To ensure that the study cohort was old enough to have a reasonable chance to develop dementia during the study period and had completed and returned the HUNT1 questionnaire assessing alcohol consumption (Q2), our sample was restricted to people born between 1905 and 1946 participating in HUNT1 and Q2. Of the 41,163 people included in the sample, 728 (1.8 %) were excluded from the fully adjusted analysis due to missing data. Hence, 40,435 people were included in the fully adjusted analysis, of which 1084 were diagnosed with dementia.

The impact of former drinking on the relationship between alcohol and dementia is an important methodological question within this field [21]. In the present study, the HUNT2 (1995–1997) material was only used to check the stability of reported alcohol consumption from HUNT1 to HUNT2. In HUNT2, 70 % of the total adult population in NT participated.

Dementia data were collected through two procedures. In both procedures, ICD-10 was used to diagnose dementia; for sub-classification, ICD-10 was used for AD and VaD, McKeith criteria for dementia with Lewy bodies, and the Manchester–Lund criteria for frontotemporal dementia. Thorough information about the HMS data collection criteria, procedures, and instruments used are described elsewhere [22].

First, during the period 2008–2010, patient files from the memory clinics at the two hospitals in NT were examined to find patients registered with a dementia diagnosis during the period 1998–2010. The use of standardised dementia diagnostic procedures was established in 1998 in these two hospitals. Before that, dementia diagnoses were rare in hospital journals. Four experts in old age psychiatry and geriatric medicine retrospectively validated the dementia diagnoses after reading hospital journals. These experts also established the age of symptom debut for each dementia patient based on hospital journal information.

Second, from 2010 to 2011, all nursing home residents in NT were invited to participate in an extensive health examination focusing on dementia diagnoses and related variables. Using standardised interviews for the assessment of cognitive decline and dementia, trained research nurses collected information from the patients’ primary nurses and primary family caregivers, including data regarding when the patient initially showed symptoms of dementia.

The final HMS sample consists of 920 patients with dementia assessed at the region’s two memory clinics and 620 patients with dementia living in nursing homes. Since 106 participants were assessed in both a memory clinic and nursing home, a total of 1434 participants were diagnosed with dementia.

Based on population figures for NT from Statistics Norway [23] and dementia prevalence estimates of the Norwegian Directorate of Health [24], we have calculated that approximately 3000 of the HUNT1, 2 and/or 3 participants may have developed dementia, whereas 1434 cases were identified by the two procedures used to assess dementia cases in NT. Among the HUNT1-Q2 participants born during 1905–1946, 1097 were diagnosed with a form of dementia: 600 with AD, 182 with VaD, 108 with mixed AD and VaD, 62 with frontotemporal dementia, 45 with Lewy body dementia, 73 with unspecified dementia, 15 with dementia caused by Parkinson’s disease, 6 with alcohol-induced dementia, and 6 for whom a subtype diagnosis could not be established. Hence, according to this estimate, nearly 50 % of possible cases of dementia in NT were identified, of whom 1097 (37 %) are included in the present sample of HUNT1-Q2 participants born during 1905–1946.

AD is a neurodegenerative disease, and the group of people diagnosed with AD in our sample is sufficiently large to be analysed separately. For both VaD and the mixed AD/VaD dementia group, different vascular illnesses are considered to be the important cause(s) of dementia. Since the numbers of cases in the two latter groups were relatively small in this study, for both conceptual and statistical reasons, VaD and mixed AD/VaD were collapsed and analysed as one group: “VaD including mixed AD/VaD.”

The Regional Committees for Medical and Health Research Ethics (REC Central Norway) approved the project.

### Measures

In HUNT1Q2, the respondents were asked to rate how often they drank alcohol during the last 14 days. The response options were as follows: 1 = I did not drink alcohol, though I am not an abstainer (classified as drinking rarely); 2 = I drank alcohol 1–4 times (classified as drinking infrequently); 3 = I drank alcohol 5–10 times; 4 = I drank alcohol more than 10 times; and 5 (recoded to 0) = I am an abstainer, I never drink alcohol. The numbers of individuals who reported either drinking 5–10 times or drinking more than 10 times within the last fortnight in HUNT1 and were later diagnosed with dementia were relatively low: 34 and 35, respectively. To ensure sufficient statistical power in the analysis involving several covariates, categories 3 and 4 were therefore collapsed and classified as drinking alcohol frequently.

The respondents in HUNT2 were asked if they were abstaining from alcohol or not. In addition, alcohol consumption frequency was numerically measured by the respondents answering the question: “How many times do you usually drink alcohol during one month?” A HUNT2 alcohol-variable was constructed to match the HUNT1 alcohol consumption variable.

Data on education (i.e., total number of years attending any kind of school) registered by Statistics Norway were categorized according to the participants’ years of education as follows: 1 = up to 7 years of education (reference group in the analysis); 2 = 8–10 years of education; 3 = 11–13 years of education; and 4 = 14 years of education or more.

Because an increasing amount of research supports the hypotheses that mid-life hypertension and obesity are risk factors for dementia [25], these variables were included as covariates in the analysis. Systolic (SBP) and diastolic blood pressure (DBP) were measured twice in HUNT1 according to a standardised method described elsewhere [20]. The second blood pressure assessment was used to categorise severe hypertension into a dichotomous (no/yes) variable using the thresholds SBP ≥ 160 and DBP ≥ 90. Body mass index (BMI) was based on height and weight measured at the health examination and calculated as weight (kg) by height (m) squared, and analysed as a dichotomised variable for obesity “no”/“yes”; 0 < 29 and 1 ≥ 30. Current or prior tobacco smoking was analysed as 0 = non-smoker, 1 = current or prior smoker, and 2 = missing values, using the responses on the two items “Do you currently smoke daily?” and “If you do not currently smoke cigarettes daily, have you ever smoked cigarettes daily?”

No validated or acknowledged depression measurement was included in HUNT1. However, a sum score of the HUNT1-items “Would you say you are usually cheerful or down spirited”, “Do you mostly feel strong and fit, or tired and worn out”, and “Thinking about your life at the moment, would you say that you by and large are satisfied with life, or are you mostly dissatisfied?” was used to assess symptoms of depression. The original score options for all three items were 1–7 (very cheerful’–‘very down spirited’, ‘very strong and fit’–‘very tired and worn out’, and ‘very satisfied’–‘very dissatisfied’, respectively). The factor analysis showed a Cronbach α = 0.74. Before being entered into the analyses, the proxy variable for symptoms of depression was dichotomised into 1 = no depressive symptoms (sum score <12), and 2 = symptoms of depression (sum score ≥12).

### Statistical analysis

SPSS 20.0 was used for the descriptive analyses and survival analyses. The relative risk for dementia, Alzheimer’s disease, and vascular dementia associated with alcohol consumption was estimated by Cox proportional hazards analysis. Since it is possible that light to moderate alcohol drinking may protect against dementia, the group “drinking infrequently” was used as a reference group. The time scale used was follow-up time in years since 1995. This year was chosen because the chance of detecting dementia cases prior to 1995 was very low. People who died before dementia assessment were censored at year of death.

The risk of dementia increases at a steeper gradient with increasing age, particularly after the age of 65 years. For this reason, and because the formula (age—45) squared fits the actual distribution of dementia across age in our data quite well, the analysis was adjusted for age at entry/baseline and transformed into a quadratic function by the formula: (age at HUNT1—45 years) [2].

We also adjusted for other variables possibly associated with increased dementia risk, including sex, education, hypertension (no/yes), smoking (no/current or former/unknown), body mass index (BMI ≥ 30), and symptoms of depression (no/yes).

The proportional hazards assumption was checked by analysis of Schoenfeld residuals using the cox.zph test procedure in the statistical software R (R Foundation for Statistical Computing, Vienna, Austria). Graphical inspection of estimated hazard function and the p-values showed no violations of the proportionality assumptions (results not shown).

To investigate the robustness of the results, sensitivity analyses were conducted by repeating the fully adjusted model stratified by narrower age cohorts: HUNT1-Q2-participants born (1) 1915–1946 and (2) 1925–1946, and by using the same time scale as in the main analysis. By excluding the oldest cohorts we reduced possible bias associated with cases left unidentified because they died before we were able to observe them.

Missing values for the alcohol consumption frequency variable (4.8 %) and smoking (2.5 %) were coded and analysed as separate categories, labeled “unknown”. Information about years of education from Statistics Norway was missing for 0.3 %. For the objectively assessed measurements of BMI (weight and height), and hypertension in HUNT1, 0.5 and 0.1 % of the values were missing, respectively. For the sum score of the three items assessing symptoms of depression, 6.6 % of the values were missing. Missing values in the depression variable were imputed by the expectation–maximization (EM)-method. Otherwise, missing data were deleted listwise.

## Results

The group later diagnosed with dementia was older (mean age 60.8 years, SD 9.0) and, as shown in Table 1, included more women (63.9 %) at baseline than the group not identified with a dementia diagnosis (mean age 57.9 years, SD 11.9, 51.2 % women). Table 2 shows that during the observation period, 61.2 % of the dementia cases and 55.4 % of the individuals in the non-dementia group died. The total number of person-years included in this study was 410,143 with a mean of 9.96 person-years.

**Table 1:** Descriptives of demographic and lifestyle factors of the sample participating in HUNT1, questionnaire 2 participants born between 1905 and 1946 (n = 1097/N = 41,163)

	n (%)	N (%)	*p* value*
Women (%)	701 (63.9)	20,498 (51.2)	<0.001
*Alcohol drinking last 14 days, HUNT1, times*			
Abstainers	182 (16.6)	5688 (14.2)	<0.001
0 times, but not abstainers	529 (48.2)	18,900 (47.2)	
1–4 times	242 (22.1)	11,182 (27.9)	
5 or more times	69 (6.3)	2400 (6.0)	
Unknown/missing	75 (6.8)	1896 (4.7)	
*Years of education*			
Until 7 years	578 (52.7)	17,029 (42.6)	<0.001
Until 10 years	75 (6.8)	4054 (10.1)	
11–13 years	367 (33.5)	14,702 (36.8)	
14 years or more	76 (6.9)	4173 (10.4)	
Current or prior smoker, yes	490 (44.7)	21,403 (53.4)	<0.001
Hypertension, yes	182 (16.6)	6852 (17.1)	0.656
BMI ≥ 30 (SD)	20 (1.8)	744 (1.9)	0.921
Symptoms of depression, yes	180 (16.4)	6313 (15.8)	0.559

n = number of cases, N = persons at risk

* *p* values are derived from *t* tests for age and χ^2^ testes for the binary variables

**Table 2:** Total number of deaths during the period 1984 to 2011 and per year between 1995 and 2011 in the study cohort participating in HUNT1-Q2 born between 1905 and 1946

	Total	1995	1996	1997	1998	1999	2000	2001	2002
n	671 (61.2*)	0	0	0	2 (0.5*)	2 (0.2*)	7 (0.6*)	6 (0.5*)	14 (1.3*)
N	22,210 (55.4*)	852 (2.3*)	926 (2.3*)	929 (2.3*)	944 (2.4*)	981 (2.4*)	1045 (2.6*)	962 (2.4*)	1013 (2.5*)

	2003	2004	2005	2006	2007	2008	2009	2010	2011
n	38 (3.4*)	42 (3.8*)	59 (5.4*)	72 (6.6*)	75 (6.8*)	50 (4.6*)	60 (5.5*)	78 (7.1*)	166 (15.1*)
N	918 (2.3*)	858 (2.1*)	862 (2.2*)	835 (2.1*)	857 (2.1*)	811 (2.0*)	764 (1.9*)	858 (2.1*)	723 (1.8*)

* % of total within each group. Dementia cases: n = 1097 and persons at risk, N = 41,163

The Spearman correlation of the alcohol consumption frequency item in HUNT1 and HUNT2 in the total sample was 0.65 and, in the group later diagnosed with dementia, 0.68, indicating that the stability of alcohol consumption between HUNT1 and HUNT2 was high. Of the respondents that reported abstaining from alcohol in HUNT1, 89 % of those also participating in HUNT2 reported abstaining from alcohol in HUNT2. Of the respondents abstaining from alcohol in HUNT2 that also participated in HUNT1, 51 % reported abstaining from alcohol, and 98 % were abstainers or “rare drinkers”, in HUNT1.

The results of the Cox regression analyses for all dementia diseases combined are shown in Table 3. Compared to reporting drinking infrequently, there was increased dementia risk when reporting abstaining from alcohol, drinking rarely, and drinking frequently in the analysis adjusted for age and sex. However, when adjusting for age, sex, education, hypertension, obesity, smoking, and symptoms of depression, only drinking frequently was significantly associated with increased dementia risk as compared to the reference group (HR 1.40, 1.07–1.84). The results of the risk analysis for Alzheimer’s diseases are shown in Table 4, and for vascular dementia in Table 5. Compared to infrequent drinking, the hazard ratios indicate a possible increased risk for Alzheimer’s disease and vascular dementia in high consumers, but this association was only statistically significant for Alzheimer’s disease (HR 1.47, 1.00–2.16). The results from the analyses stratified by age indicate the same trends as in the main analysis for the age group 60 years or older at time of participation in HUNT1. As shown in Table 6, the results of the sensitivity analyses indicate that the patterns of associations resemble the results of the main analysis. None of the hazards ratios in the age stratified samples reached statistical significance.

**Table 3:** Alcohol consumption at HUNT1 and the association with dementia (one group) up till 27 years later

	Hazard ratios (95 % Confidence Intervals, CI) by alcohol consumption last 14 days			
	Abstainers	Drinking 0 times, not abstainers	Drinking 5 or more times	Unknown
Crude	2.06 (1.69–2.51) *p* < 0.001	1.59 (1.36–1.85) *p* < 0.001	1.57 (1.20–2.05) *p* = 0.001	3.13 (2.40–4.10) *p* < 0.001
Adjusted for age^b^, sex	1.30 (1.05–1.61) *p* = 0.015	1.22 (1.04–1.43) *p* = 0.017	1.45 (1.11–1.90) *p* = 0.007	1.73 (1.30–2.29) *p* < 0.001
Fully adjusted model^a^	1.15 (0.92–1.43) *p* = 0.223	1.12 (0.95–1.32) *p* = 0.181	1.40 (1.07–1.84) *p* = 0.015	1.52 (1.13–2.05) *p* = 0.006

Cox survival analysis. HUNT1Q2-participants born between 1905 and 1946 (n = 1084/N = 40,435)

Reference group: respondents reporting to drink alcohol 1–4 times the last fortnight

^a^Adjusted for age, ^b^ (=ageHUNT1-45)^2^, sex, years of education, hypertension (≥160/90), BMI ≥ 30, current or prior smoking and symptoms of depression (no/yes)

**Table 4:** Alcohol consumption at HUNT1 and the association with Alzheimer’s disease up till 27 years later

	Hazard ratios (95 % Confidence Intervals, CI) alcohol consumption last 14 days			
	Abstainers	Drinking 0 times, not abstainers	Drinking 5 or more times	Unknown
Alzheimer’s disease, crude	2.21 (1.68–2.91) *p* < 0.001	1.86 (1.50–2.31) *p* < 0.001	1.61 (1.10–2.36) *p* = 0.015	4.02 (2.83–5.71) *p* < 0.001
Alzheimer’s disease, adjusted for age^b^, sex	1.26 (0.94–1.69) *p* = 0.127	1.34 (1.07–1.68) *p* = 0.010	1.52 (1.03–2.22) *p* = 0.033	1.99 (1.38–2.88) *p* < 0.001
Alzheimer’s disease, fully adjusted model^a^	1.09 (0.80–1.48) *p* = 0.595	1.20 (0.96–1.51) *p* = 0.114	1.47 (1.00–2.16) *p* = 0.048	1.73 (1.17–2.55) *p* = 0.006

Cox survival regression analysis. HUNT1Q2-participants born between 1905 and 1946 (n = 595/N = 40,435)

Dementia group included Alzheimer cases only. Other dementia cases in these analyses were censored. Reference group: Respondents reporting to drink alcohol 1–4 times the last fortnight

^a^ adjusted for age, ^b^ (ageHUNT1-45)^2^, sex, age, education, severe hypertension (≥ 160/90), BMI ≥ 30, smoking and symptoms of depression

**Table 5:** Alcohol consumption at HUNT1 and the association with vascular dementia (VaD) including mixed VaD/VD up till 27 years later

	Hazard ratios (95 % Confidence Intervals, CI) alcohol consumption last 14 days			
	Abstainers	Drinking 0 times, not abstainers	Drinking 5 or more times	Unknown
	1.89 (1.30–2.74) *p* = 0.001	1.29 (0.96–1.73) *p* = 0.091	1.63 (1.00–2.65) *p* = 0.050	3.06 (1.86–5.03) *p* < 0.001
Vascular dementia including mixed AD/VD adjusted for age^b^, sex	1.24 (0.83–1.84) *p* = 0.298	1.01 (0.74–1.37) *p* = 0.951	1.52 (0.93–2.47) *p* = 0.095	1.76 (1.04–2.97) *p* = 0.033
Vascular dementia including mixed AD/VD Fully adjusted model^a^	1.15 (0.75–1.76) *p* = 0.520	0.96 (0.71–1.32) *p* = 0.817	1.43 (0.87–2.36) *p* = 0.161	1.48 (0.85–2.61) *p* = 0.167

HUNT1Q2-participants born between 1905 and 1946 (n = 284/N = 40,435)

Dementia group included vascular and mixed (AD/VD) dementia cases only. Other dementia cases in these analyses were censored. Reference group: Respondents reporting to drink alcohol 1-4 times the last fortnight

^a^adjusted for age, ^b^ (ageHUNT1-45)^2^, sex, age, education, severe hypertension (≥ 160/90), BMI ≥ 30, smoking and symptoms of depression

**Table 6:** Sensitivity analyses including overall dementia

	Hazard ratios (95 % Confidence Intervals, CI) by alcohol consumption last 14 days			
HUNT1Q2-participants born between 1915 and 1946	Abstainers	Drinking 0 times, not abstainers	Drinking 5 or more times	Unknown
	1.07 (0.85–1.34) *p* = 0.584	1.04 (0.88–1.24) *p* = 0.613	1.32 (1.00–1.75) *p* = 0.052	1.33 (0.97–1.82) *p* = 0.074
HUNT1Q2-participants born between 1925 and 1946	Abstainers	Drinking 0 times, not abstainers	Drinking 5 or more times	Unknown
HUNT1Q2-participants born between 1905 and 1946	0.76 (0.53–1.10) *p* = 0.152 Abstainers	0.89 (0.71–1.12) *p* = 0.329 Drinking 0 times, not abstainers	1.30 (0.88–1.90) *p* = 0.177 Drinking 5 or more times	1.12 (0.66–1.91) *p* = 0.670 Unknown
	1.20 (0.97–1.49) *p* = 0.090	1.12 (0.95–1.32) *p* = 0.152	1.38 (1.06–1.81) *p* = 0.018	1.60 (1.20–2.13) *p* = 0.001

Results from fully adjusted analyses^a^

Reference group: respondents reporting to drink alcohol 1-4 times the last fortnight

^a^ Adjusted for age, ^b^ (=ageHUNT1-45)^2^, sex, years of education, severe hypertension (≥160/90), BMI ≥ 30, current or prior smoking and symptoms of depression (no/yes)

## Discussion

The present study finds that frequent alcohol consumption is associated with a higher dementia risk compared to infrequent alcohol consumption. The same pattern of associations is indicated for Alzheimer’s disease and for vascular dementia, but the latter results were not statistically significant.

Previous research consistently reports that heavy drinking is associated with increased dementia risk [11]. An average intake of a couple of alcoholic drinks per day, and perhaps only one drink per day for women, may increase the dementia risk [17]. On the contrary, several studies assessing alcohol use among elderly have found that light to moderate alcohol consumption is associated with reduced risk for dementia [5, 16, 18, 26]. A meta-analysis by Anstey et al. [7] reported a 25–28 % estimated reduction in risk for any dementia associated with light to moderate alcohol intake compared to alcohol abstinence in older adults, but the causality was uncertain [11]. Using a relatively young sample (mean age of 58 years) at the time of the alcohol consumption assessment and long observation period, the present study does not support the hypothesis that infrequent alcohol consumption is associated with reduced risk of dementia compared to abstaining from alcohol. The previously reported associations between light to moderate alcohol consumption and reduced dementia risk could reflect better physical, dietary, and mental health in the group of moderate drinkers as compared to the abstainers. Such possible group difference in health could be less evident in our study, involving a sample that was much younger at the time of the alcohol assessments.

Some limitations of this study should be pointed out. Given that dementia was only assessed from 1998, people born in 1905 had to be 93 years of age before dementia was first diagnosed, which makes the oldest age categories prone to survival bias. The group for whom we do not know the level of alcohol consumption, consistently in all the analyses, has the highest dementia risk. Hence, attempts to interpret why people in the “unknown alcohol consumption group” have not reported their alcohol consumption and why this group has an increased dementia risk will merely be speculations. Another limitation of the present study is that we have not examined the effect of heavy drinking. In addition, relatively few participants (6.5 %) reported drinking alcohol frequently (five times or more) during the last 14 days. This may have somewhat affected the statistical power of the analyses.

Estimated yearly alcohol consumption in Norway in the eighties was around 7 l pure alcohol per citizen [27] corresponding to a mean consumption of around seven units per 14 days. Accordingly, on average the respondents have probably underreported the number of drinks due to the stigma often associated with alcohol consumption and alcohol abuse. Such an under-reporting does not necessarily imply that the rank-order of intake in the alcohol frequency measurement is severely affected if the degree of under-reporting is highly correlated with the true consumption. Nevertheless, it is a limitation to this study that we have no true measure of amount of alcohol consumed but have to rely on what is probably under-reported self-reports of consumption, making comparison with other studies, using other operationalisation of alcohol consumption, difficult. On the contrary, the distribution in our sample corresponds well with the distribution normally observed in alcohol consumption research at the time of HUNT1, in which approximately 2/3 reports drinking less than average consumption and 15 % more than twice the average [28].

Some uncertainty is associated with the fact that many HUNT1 participants died before our observation period without being registered as having dementia, possibly introducing some bias due to “competing risk”. One example is that a disproportionally large number of heavy alcohol users may have died before the observation period, and thus were lost as observed cases. If abstainers are generally less healthy than moderate consumers, the same may have been the case for this group. Increased mortality in these groups may have deflated the risk estimates for frequent drinking as well as for abstaining from alcohol. Excluding the oldest part of the population is expected to reduce such a bias if present. The similar results for the age-stratified samples as for the full sample indicate that such a bias has not dramatically affected the results.

Not all respondents in HUNT1 who later developed dementia, have been discovered and registered in HMS. Hence, some of the individuals included in the comparison group may have dementia. However, the inclusion of individuals with dementia in the large comparison group would not markedly affect the risk estimations, but might lead to moderate underestimations. The strengths of this study comprise the high response rate of HUNT1, the long period from observed exposure to observed outcome, and the fact that the sample included a substantial number of people with a verified dementia diagnosis. Since drinking alcohol is common in many parts of the world and the life expectancy in most populations is increasing, this study addresses an internationally important public health issue.

The balance between healthy and troublesome levels of alcohol consumption is difficult to define. A recent study investigating 140 neuropsychologists’ beliefs about alcohol and dementia found that almost all of the respondents (93 %) reported that alcohol has neurotoxic effects, and most of them were not sure whether moderate alcohol consumption can be protective [29]. Our results are in line with most previous research finding that frequent alcohol consumption increases the risk for dementia compared to infrequent alcohol consumption. The present analysis indicates that abstaining from alcohol may be associated with increased dementia risk when adjusted for age and sex. However, this relationship is no longer present when adjusting for other somatic and mental health factors associated with increased dementia risk.
